# Preoperative predictive factor analysis of ovarian malignant involvement in premenopausal patients with clinical stage I endometrioid endometrial carcinoma

**DOI:** 10.1038/s41598-020-78953-4

**Published:** 2021-01-13

**Authors:** Qin Chen, Yan Feng, Wenwen Wang, Weiguo Lv, Baohua Li

**Affiliations:** 1 Department of Pathology, Women’s Hospital, Zhejiang University School of Medicine, Xueshi Road 1, Hangzhou, 310006 Zhejiang People’s Republic of China; 2 Department of Gynecologic Oncology, Women’s Hospital, Zhejiang University School of Medicine, Xueshi Road 1, Hangzhou, 310006 Zhejiang People’s Republic of China; 3 Center of Uterine Cancer Diagnosis & Therapy of Zhejiang Province, Xueshi Road 1, Hangzhou, 310006 Zhejiang People’s Republic of China

**Keywords:** Cancer, Biomarkers, Risk factors

## Abstract

Earlier literature suggests that ovarian preservation in young premenopausal clinical stage I endometrioid endometrial carcinoma patients does not negatively impact prognosis. The main purpose of this study was to clarify the incidence of ovarian malignant involvement in this group and further identify potential preoperative predictive factors of ovarian malignant involvement. A total of 511 premenopausal (age ≤ 50 years) patients were enrolled for the study at Women’s Hospital, Zhejiang University School of Medicine, between January 2002 and December 2016. Ovarian malignant involvements were detected in 23 of the patients (4.5%). Univariate and multivariate logistic analysis validated preoperative imaging of myometrial invasion depth and preoperative serum carbohydrate antigen 125 (CA125) level as independent risk predictors of postoperative ovarian malignant involvement. Receiver operating characteristic (ROC) curves was generated for a combination of the two factors. The area under curve (AUC) was 0.772 (95% confidence interval [CI] 0.661–0.884) for the combined two factors. The incidence of postoperative ovarian malignant involvement was relatively minimal. Preoperative imaging of myometrial invasion depth and serum CA125 level were independent risk predictors of ovarian malignant involvement. These findings may facilitate preoperative counseling of patients and informed clinical decision-making on ovarian preservation in these patients.

## Introduction

Endometrial carcinoma is one of the most common gynecological malignancies prevalent in postmenopausal females in the sixth and seventh decades of life^1^. In 2012, the number of new cases and deaths due to endometrial cancer worldwide was 319,605 and 76,160 respectively^2^. The median age at diagnosis is 61 years. At present, the incidence of young premenopausal women with endometrial carcinoma is gradually increasing, with 2–14% women aged < 40 years^3^ and 5–30% women aged ≤ 50 years at the time of diagnosis^4^. The standard surgical approach for endometrial carcinoma includes total hysterectomy and bilateral salpingo-oophorectomy (BSO) with or without pelvic and/or para-aortic lymph node dissection, according to the existence of high-risk factors^1,5,6^. In the majority of young premenopausal patients, endometrioid endometrial carcinoma is the most common endometrial carcinoma subtype and is usually confined to clinical stage I, namely, located within the corpus uteri without malignant involvement extending beyond the uterus and metastasis to the pelvic and/or para-aortic lymph nodes^4,7^.

Oophorectomy is commonly performed in young premenopausal women with endometrioid endometrial carcinoma in conjunction with hysterectomy, as an estrogen-responsive tumor. In terms of therapeutic benefits, oophorectomy not only decreases estrogen production but also eliminates the occult co-existing involved ovarian metastasis. However, a surgical pathological study for clinical stage I endometrial carcinoma conducted by the Gynecologic Oncology Group reported only a 5% incidence of adnexal involvement, especially in young premenopausal women^8^. The incidence of isolated microscopic ovarian involvement was also uncommon (~ 1%)^9^. In addition, estrogen deprivation resulting from oophorectomy in young premenopausal women may cause a rapid decline in circulating ovarian estrogen and androgens, leading to increased short- and long-term adverse outcomes, including hot flushes, vaginal atrophy, sleep disorders, cardiovascular disease, osteoporosis, dementia, cognitive impairment, depression and anxiety as well as permanent loss of fertility^6^. Use of this traditional surgical approach is controversial due to occasional ovarian involvement and adverse sequela of estrogen deprivation in young premenopausal patients with clinical stage I endometrioid endometrial carcinoma. However, the safety and feasibility of ovarian preservation in the patient group is of widespread concern. To our knowledge, limited studies have focused on preoperative predictive factors of ovarian malignant involvement in these patients, which may be utilized to determine the optimal surgical approaches for providing maximal therapeutic benefits and further distinguish patients requiring BSO.

Here, we performed a retrospective study on premenopausal patients with clinical stage I endometrioid endometrial carcinoma admitted in Women’s Hospital, Zhejiang University School of Medicine, between 2002 and 2016. The incidence of ovarian malignant involvement in these patients and possible preoperative predictive factors were further determined, with a view to optimizing therapeutic management.

## Methods

This retrospective study was performed on premenopausal patients (age ≤ 50 years) with clinical stage I endometrioid endometrial carcinoma subjected to total hysterectomy and BSO with or without pelvic and/or para-aortic lymph node dissection from Women’s Hospital, Zhejiang University School of Medicine, between January 2002 and December 2016. Patients were identified using hospital tumor registries and an internal database of gynecological carcinoma cases. During this period, 1978 patients, who were diagnosed with endometrial carcinoma, were admitted in our hospital. CT/MRI imaging was performed to examine the size and location of uterine tumors, myometrial invasion depth and the presence or absence of enlarged or suspicious paraortic and pelvic nodals as well as adnexal involvement. Basis on the preoperative imaging results, patients with suspicious paraortic and pelvic nodal or adnexal involvement were all excluded. The patients were also excluded with family history of colon or gastrointestinal carcinoma. Finally, the included patients with clinical stage I endometrioid endometrial carcinoma were 1228 cases. Among them, the numbers of premenopausal patients (age ≤ 50 years) were 522. Basically, the enrolled patients did not receive preoperative adjuvant treatment, on account of premenopausal clinical stage I endometrioid endometrial carcinoma management option.

All pathological sections were confirmed by two gynecological pathologists (QC and WWW). Ovarian malignant involvement was further determined via postoperative pathologic examination. Among the 522 patients examined, 34 displayed ovarian malignancy involvement, specifically, ovarian endometrioid carcinoma (23 cases), synchronous primary uterine and ovarian tumors (4 cases), borderline tumor (3 cases), serous papillary carcinoma (2 cases), clear cell carcinoma (1 case) and mucinous adenocarcinoma (1 case). Patients with endometrioid histology of ovary and uterine were included, while other pathological subtypes and independent synchronous primary uterine and ovarian tumors were excluded, according to the pathological criteria of Ulbright and Roth in 1985^10^ used to distinguish ovarian malignant involvement from independent synchronous endometrial and ovarian carcinoma. Furthermore, an extensive pathological characteristic formulated by Scully et al.^11^ was applied to differentiate between uterine endometrial carcinoma with ovarian malignant involvement and independent synchronous primary uterus and ovarian carcinoma. Clinicopathological characteristics favoring primary uterine endometrial tumors with ovarian malignant involvement included histological similarity of tumors, uterine larger size of tumor, presence of uterine atypical endometrial hyperplasia and deep myometrial invasion with direct extension into adnexa and/or lymphovascular invasion. And other evidence of spread from uterine endometrial tumor included bilateral, multinodular, surface involvement of the ovary and absence of endometriosis^11^. Finally, 511 premenopausal patients (≤ 50 years) with clinical stage I endometrioid endometrial carcinoma were selected for statistical analysis, according to the above admittance standards. Patient demographics and clinicopathological data were obtained through hospital electronic medical records systems and paper charts (Table 1).

**Table 1:** Univariate logistic analysis showed the preoperative predictive factors of ovarian malignant involvement in 511 premenopausal patients with clinical stage I endometrioid endometrial carcinoma.

Characteristics	Ovarian malignant involvement, n (%)		*P*-value
	No	Yes	
**Patient age (year)**			0.671
	43.93 ± 5.52	43.26 ± 7.41	
**Preoperative BMI (kg/m**^**2**^**)**			0.381
< 30	443(86.7)	22(4.3)	
≥ 30	45(8.8)	1(0.2)	
**Preoperative pathological tumor grade**			0.158
Well/moderate	463(90.6)	20(3.9)	
Poor	25(4.9)	3(0.6)	
**Tumor in specific-site**			0.422
No	449(87.9)	20(3.9)	
Yes	39(7.6)	3(0.6)	
**Preoperative myometrial invasion depth**			1.0E^-6^
< 1/2	466(91.2)	14(2.7)	
≥ 1/2	22(4.3)	9(1.8)	
**Preoperative tumor size (cm)**			0.111
< 4	407(79.6)	16(3.1)	
≥ 4	81(15.9)	7(1.4)	
**Peritoneal lavage cytology**			0.107
Negative	486(95.1)	22(4.3)	
Positive	2(0.4)	1(0.2)	
**Preoperative serum CA125 (U/L)**			3.2E^-5^
	22.65(0.4–392)	135.2(1.0–834)	

*BMI* Body mass index, *CA125* Carbohydrate antigen 125.

All 511 patients were divided into low risk (n = 193) and sub-high risk groups (n = 318) according to the degree of endometrioid endometrial cancer differentiation and/or (≥ 1/2) myometrial invasion^12^. Preoperative clinicopathological factors were used to evaluate the likelihood of postoperative ovarian malignancy involvement.

All 511 patients enrolled in the study were followed up postoperatively by interview at the clinic or telephone call. Disease-free survival (DFS) and overall survival (OS) rates were calculated from the day of the surgery until recurrence or death. The deadline of follow-up was December 2018. In total, 89 patients (89/511, 17.42%) were lost to follow-up. The mean follow-up time was 94.56 months (range 12–202 months). We recorded 26 recurrences (26/422, 6.16%) and 22 deaths (22/422, 5.21%) during this period.

Statistical analysis was performed using SPSS version 23.0. Kaplan–Meier analysis was used to evaluate disease-free and overall survival rate differences between patients with and without ovarian malignant involvement. We applied univariate and multivariate regression models to analyze preoperative predictive factors for ovarian malignant involvement in clinical stage I endometrioid endometrial carcinoma and plotted receiver operating characteristic (ROC) curves. Data were considered significant at *p*-values < 0.05.

The present study was approved by the Institutional Review Board of Women’s Hospital. Informed consent was obtained from all individual participants.

### Ethics approval and consent to participate

The present study was approved by the Institutional Review Board of Women’s Hospital, Zhejiang University School of Medicine. Written, informed consent has been given and obtained from all individual participants included in the study. All methods were carried out in accordance with relevant guidelines and regulations.

## Results

A total of 511 premenopausal (≤ 50 years) patients with clinical stage I endometrioid endometrial carcinoma were identified. Among these, 28 patients were poorly differentiated according to preoperative pathological data by endometrial biopsy, and 31 cases had deep myometrial invasion (≥ 1/2) by preoperative imaging examination (Table 1). Furthermore, 193 patients from low risk groups were subjected to total hysterectomy and BSO. A further 318 patients identified with at least one risk factor of sub-high risk groups underwent total hysterectomy and BSO with pelvic and/or para-aortic lymph node dissection.

According to postoperative clinicopathological data (Table 2), lymph vascular space invasion involvement (LVSI) was identified in 15 (2.94%) of 511 cases; lymph node metastasis (LNM) was confirmed in 16 (5.03%) of 318 cases; 41 cases were poorly differentiated; 34 cases had deep myometrial invasion (≥ 1/2). More importantly, the overall ratio of ovarian malignant involvement in 511 patients with clinical stage I endometrioid endometrial carcinoma was 4.5% (23/511). Among them, 6 cases presented isolated ovarian metastases, 16 cases with the diameter of 0.5–1 cm ovarian metastases and 7 with the diameter less than 0.5 cm microscopic ovarian metastases, affirmed by the final pathological diagnosis. The counterparts of low risk and sub-high risk patient groups were 1.04% (n = 2) and 6.6% (n = 21). The sub-high risk group was associated with a higher rate of ovarian malignancy involvement (*P* = 0.003) than the low-risk group.

**Table 2:** Postoperative demographics and clinic pathological data of 511 premenopausal patients with clinical stage I endometrioid endometrial carcinoma.

Characteristics	No. of patients	Percentage (%)
**Patient age (year)**		
≤ 40	121	23.68
> 40 and ≤ 50	390	76.32
**FIGO stage**		
< II	384	75.15
≥ II	127	24.85
**Differentiation**		
Well	363	71.04
Moderate	107	20.94
Poor	41	8.02
**Myometrial invasion**		
< 1/2	477	93.34
≥ 1/2	34	6.65
**Lymphadenectomy**		
Yes	318	62.23
No	193	37.77
**LNM**		
Yes	16	5.03
No	302	94.97
**Cervical stromal involvement**		
Yes	92	18.01
No	419	81.99
**LVSI**		
Yes	15	2.94
No	496	97.06
**Ovarian involvement**		
Yes	23	4.50
No	488	95.50

*FIGO* International Federation of Gynecology and Obstetrics, *LVSI* lymph vascular space invasion, *LNM* lymph node metastasis.

**Figure 1:**
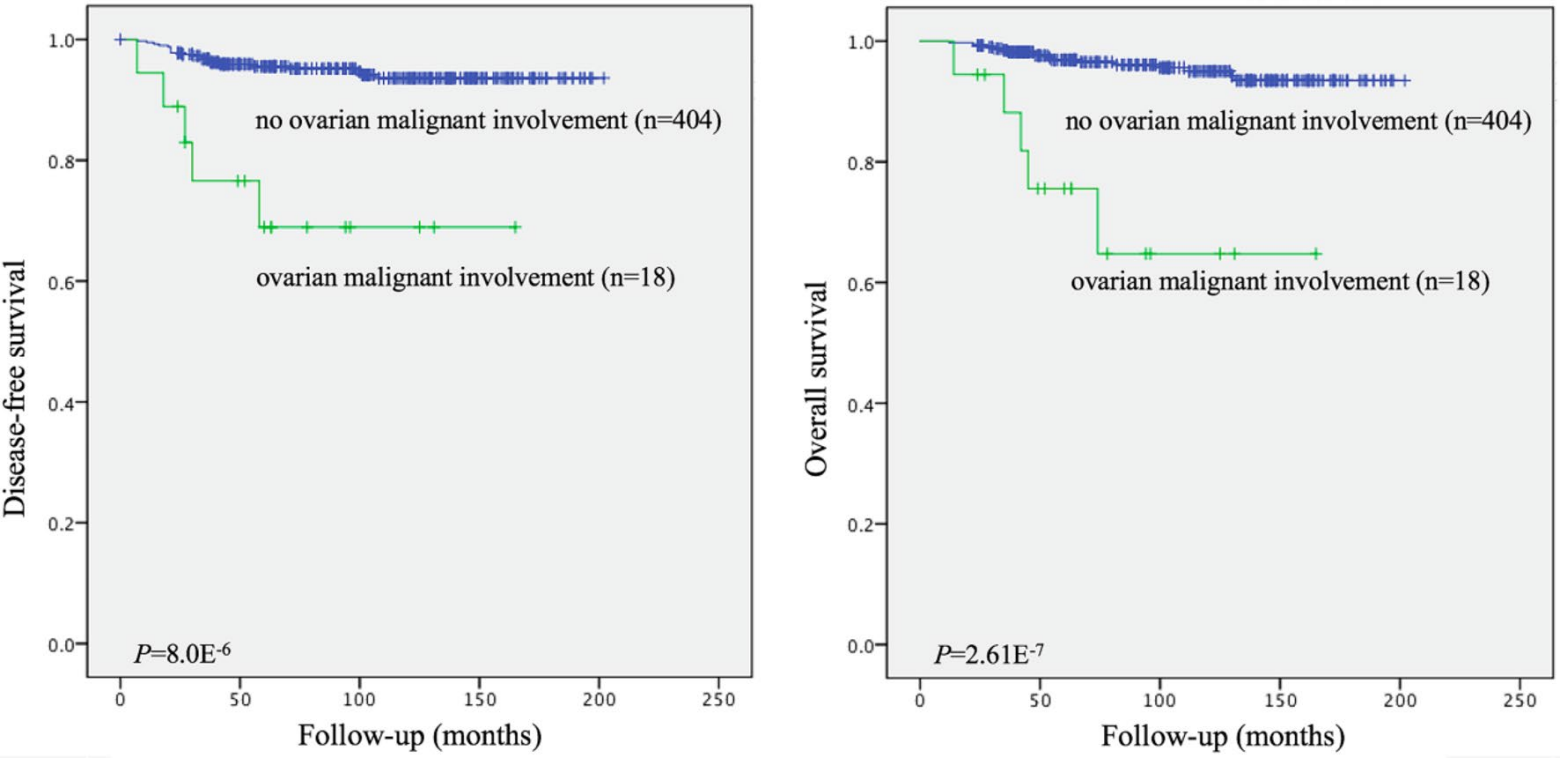
Kaplan–Meier curves showing the relationship between status of ovarian malignant involvement and disease-free survival (DFS) and overall survival (OS) in 422 premenopausal patients with clinical stage I endometrioid endometrial carcinoma. The patients with ovarian malignant involvement were significantly associated with shorter DFS (**A**) and OS (**B**).

In Kaplan–Meier analysis, patients without ovarian malignant involvement displayed longer disease-free survival (*P* = 8.0E^−6^, Fig. 1A) and higher 5-year survival rates (*P* = 2.61E^−7^, Fig. 1B) than those with ovarian malignant involvement. Differences were significant between two groups, clearly indicating poorer prognosis of patients with ovarian malignant involvement.

Next, univariate and multivariate regression models were applied to determine preoperative predictive factors for ovarian malignant involvement in clinical stage I endometrioid endometrial carcinoma patients. Univariate logistic analysis disclosed the correlations of preoperative imaging of myometrial invasion depth (*P* = 1.0E^−6^) and serum Carbohydrate Antigen 125 (CA125) level (*P* = 3.2E^−5^) with prediction of ovarian malignant involvement in these patients (Table 1). Multivariate logistic analysis further validated preoperative imaging of myometrial invasion depth (*P* = 0.005) and preoperative serum CA125 level (*P* = 2.51E^−4^) as independent risk predictors of ovarian involvement (Table 3). Other preoperative risk factors, including age at diagnosis, preoperative BMI, preoperative pathological tumor grade, tumor in specific-site, preoperative tumor size and peritoneal lavage cytology, did not appear associated with ovarian malignant involvement.

**Table 3:** Multivariate logistic analysis showed the preoperative predictive factors of ovarian malignant involvement in 511 premenopausal patients with clinical stage I endometrioid endometrial carcinoma.

Characteristics	Regression coefficient (B)	Standard error (SE)	χ^2^ (Wald)	*P*-value
Preoperative myometrial invasion depth	1.099	0.395	7.763	0.005
Preoperative serum CA125 (U/L)	0.014	0.004	13.405	2.51E^−4^

*CA125* Carbohydrate antigen 125.

**Figure 2:**
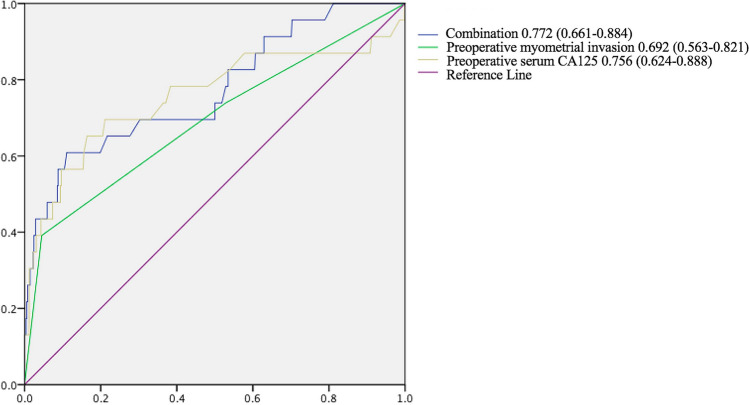
AUC of the multivariate base model (the combination of preoperative myometrial invasion depth and preoperative serum CA125 level) could have a medium intensity predictive value for postoperative ovarian malignant involvement in 511 premenopausal patients with clinical stage I endometrioid endometrial carcinoma.

Finally, ROC curves were generated with preoperative myometrial invasion depth, preoperative serum CA125 level and a combination of the two factors. The area under curve (AUC) were 0.692 (95% confidence interval [CI] 0.563–0.821) for preoperative myometrial invasion depth alone group, 0.756 (95% confidence interval [CI] 0.624–0.888) for preoperative serum CA125 level alone group and 0.772 (95% confidence interval [CI] 0.661–0.884) for the combination group, respectively. The combination of preoperative myometrial invasion depth and preoperative serum CA125 level (Fig. 2) displayed a medium intensity predictive value for postoperative ovarian malignant involvement in premenopausal clinical stage I endometrioid endometrial carcinoma patients.

## Discussion

A considerable proportion of premenopausal women are diagnosed with endometrioid endometrial carcinoma, the majority of who are classified as preoperative clinical stage I^4^. Younger premenopausal patients with clinical I stage endometrioid endometrial cancer have a more favorable prognosis than postmenopausal patients. An earlier large-scale study reported a 5-year disease-specific survival rate of 93% in women younger than 40 years^13^. The higher survival of younger patients may be partly attributed to early-stage and low-grade tumors.

For patients with clinical stage I endometrioid endometrial carcinoma, the traditional surgical approach is total hysterectomy and BSO with or without pelvic and/or para-aortic lymph node dissection according to the existence of high-risk factors. Young premenopausal women with clinical stage I endometrioid endometrial carcinoma subjected to total hysterectomy and BSO often not only suffer from permanent loss of fertility but also experience climacteric symptoms of decreased estrogen production. Hence, ovarian preservation is a feasible option for this group of patients in the absence of high risk factors. Wright and co-workers confirmed the safety and feasibility of ovarian preservation in a population-based analysis^14^. Consistently, Lee et al*.*^9,15^ reported that ovarian preservation in premenopausal women with early-stage endometrial carcinoma was not associated with poorer outcomes. Another study by Richter and colleagues showed that BSO not only induced better disease-free survival but also did not affect overall survival in young endometrial carcinoma patients^16^. Jia and Zhang recently performed a meta-analysis of comparing overall survive between BSO group (9376 cases) and ovary preservation group (1340 cases) in 10716 patients from four studies from the USA, Korea, and China. They demonstrated that ovary preservation can significantly improve the overall survive in the long run, and did not adversely impact the reduced recurrence-free survival in pre-menopausal patients with early-stage endometrial cancer^3^.

However, occasionally these patients present with occult ovarian malignant involvement, as evident from postoperative detailed pathological inspection. In our analysis, ovarian malignant involvement occurred in 4.5% patients with clinical stage I endometrioid endometrial carcinoma, in keeping with Lin et al*.*^17^ who reported ovarian involvement in 5% of their patient group. The ratio of ovarian malignant involvement in low risk patients was 1.04% while patients in the sub-high risk group positive for at least one risk factor showed higher ovarian involvement (6.6%). Data from Kaplan–Meier analysis in the current study showed longer disease-free and higher overall survival rates in patients without ovarian malignant involvement relative to those with ovarian involvement. Ovarian malignant involvement, representing an advanced stage of disease, is generally associated with poorer prognosis. Effective ways to identify patients at higher risk of ovarian malignant involvement and preoperative risk factors that can be used to predict ovarian malignant involvement, especially to distinguish the subgroups of patients suitable for receiving staging surgery for endometrioid endometrial cancer, remains an urgent requirement. The majority of studies to date have focused on postoperative pathological prediction factors, such as myometrial invasion, tumor size, lymphovascular involvement, tumor grade, lymph node metastasis and cervical invasion. Sun et al. reported adnexal morphology, lymph node involvement (confirmed via frozen sections) and tumor spread in the peritoneal cavity as the most significant predictors of ovarian involvement, based on data from 203 young women with early-stage endometrial cancer^6^. Chen et al.^18^ utilized postoperative histological and pathologic data (tumor size, histological type, pathological grade and invasive depth of myometrium, uterine serosal involvement, lymph vascular space invasion, cervical involvement, and adnexa involvement) as effective parameters for distinguishing synchronous primary cancers of endometrium and ovary and endometrial cancer with metastasis to the adnexa. Clinicopathological characteristics (i.e., tumor size, myometrial invasion, lymphovascular space involvement, lymph node metastasis, tumor grade, cervical invasion and ovarian enlargement ≥ 5 cm) were used to assess the likelihood of ovarian malignancy by Yoshino and colleagues, who showed the presence of ovarian metastasis in 4.5% patients and identified lymph node metastasis and deep myometrial invasion as significant predictive factors for ovarian metastasis and lymph node metastasis, respectively^19^. Li et al.^20^ highlighted specific post-operation parameters, such as deeper myometrial invasion, positive lymph node metastasis, positive LVSI, and high histologic grade (G2–G3), associated with ovarian involvement in younger endometrial cancer patients. Furthermore, in multivariate analysis, only deep myometrial invasion was an independent risk factor for ovarian involvement. However, this information is meaningless, because all the above risk factors were evaluated following the operation and not useful for gynecologists prior to surgery. Here, we focused on identifying potential preoperative predictive factors of ovarian malignant involvement, with the aim of providing beneficial guidelines for the appropriate surgical interventions.

Our results suggested that preoperative imaging of myometrial invasion depth and serum CA125 were predictive risk factors of ovarian malignant involvement in premenopausal clinical stage I endometrioid endometrial carcinoma patients, which could aid in preoperative counseling of patients and clinical decision-making for the first time. Univariate and multivariate logistic analyses further validated preoperative imaging of myometrial invasion depth and serum CA125 as independent risk predictors of ovarian involvement in our patient group. Consistently, AUC data showed that combination of preoperative myometrial invasion depth and serum CA125 had a medium predictive value for postoperative ovarian malignant involvement to a degree. Preoperative deeper myometrial invasion depth and serum CA125 have been previously identified as prognostic factors in ovarian metastasis^21^. Jiang and colleagues reported that preoperative serum CA125 is an effective predictor of lymph node metastasis in patients with endometrial cancer, in particular, clinical stage I^22^. Analysis of the combined factors also revealed utility as a predictive marker of ovarian malignant involvement to some extent. CA125 has been applied as a tumor marker of ovarian carcinoma since its discovery 30 years ago^23^. A large proportion (80%) of women with primary epithelial ovarian carcinoma and secondary ovarian tumor (70%) are diagnosed based on elevated CA125 levels^24,25^. Moreover, Reijnen et al. performed a prospective cohort study of women diagnosed with endometrial cancer at 9 hospitals in the Netherlands. They found the CA-125 elevated in 26.2% of women with grade 1 or 2 endometrioid endometrial cancer. Elevated CA-125 was significantly associated with advanced stage and deep myometrial invasion, and in the multivariable analysis adjusting for covariates, CA-125 was independently associated with both disease-free survival^26^. Combination of serum CA125 and preoperative myometrial invasion depth may thus present an effective predictive risk factor of postoperative ovarian malignant involvement. Other preoperative risk factors, including age at diagnosis, tumor size^19^, peritoneal lavage cytology^27^, have no predictive value for ovarian metastasis, the same results with our research.

Our study had several of limitations that should be acknowledged. One significant factor was the origin of ovarian tumor. Although we set stricter clinic pathological criteria for diagnosis, classification of a part of patients into ovarian malignant involvement or simultaneous uterine and ovarian carcinoma groups was difficult. Moreover, precursor lesions, such as endometrial hyperplasia or concurrent ovarian endometriosis, were not consistently addressed in pathology reports, potentially affecting the final diagnosis. Therefore, novel powerful genetic tools require development for accurate classification of patients displaying complex symptoms in forthcoming research, which would lead to a more accurate research database. Second, large-scale prospective clinical studies are necessary to ascertain whether the benefits of ovarian preservation outweigh the risks of surgical procedures in clinical stage I endometrioid endometrial carcinoma to reduce bias.

## Conclusions

The main purpose of the present study was to identify preoperative predictive factors of ovarian malignant involvement in premenopausal patients with clinical stage I endometrioid endometrial carcinoma. Our results showed that the incidence of ovarian malignant involvement in these patients is relatively minimal. A combination of preoperative myometrial invasion depth and serum CA125 level appeared to have predictive value for postoperative ovarian malignant involvement and may thus aid in informed decision-making on whether or not ovarian preservation should be performed in premenopausal patients with clinical stage I endometrioid endometrial carcinoma.

## Data Availability

All data generated and /or analyzed during the current study are available from corresponding author on reasonable request.

## Abbreviations

FIGO: International Federation of Gynecology and Obstetrics
LVSI: Lymph vascular space invasion
LNM: Lymph node metastasis
BMI: Body Mass Index
CA125: Carbohydrate antigen 125
DFS: Disease-free survival
OS: Overall survival
BSO: Bilateral salpingo-oophorectomy
ROC curve: Receiver operating characteristic curve
AUC: Area under curve
TPR: True positive rate
TNR: True negative rate
PPV: Positive predictive value
NPV: Negative predictive value
